# Protocol of OGSG 1901: a phase II trial of ramucirumab plus irinotecan for patients with early relapsed gastric cancer during or after adjuvant docetaxel plus S − 1 therapy

**DOI:** 10.1186/s12885-022-09844-2

**Published:** 2022-07-15

**Authors:** Toshifumi Yamaguchi, Hisato Kawakami, Daisuke Sakai, Yukinori Kurokawa, Toshio Shimokawa, Masahiro Goto, Taroh Satoh

**Affiliations:** 1 Department of Frontier Science for Cancer and Chemotherapy, Osaka University Graduate School of Medicine, Suita, Japan; 2 Department of Medical Oncology, Kindai University Faculty of Medicine, 377-2, Ohno-higashi, Osaka-sayama, Osaka, 589-8511 Japan; 3 Department of Gastroenterological Surgery, Osaka University Graduate School of Medicine, Osaka, Japan; 4 Clinical Study Support Center, Wakayama Medical University Hospital, Wakayama, Japan; 5 Cancer Chemotherapy Center, Osaka Medical College, Osaka, Japan

**Keywords:** Gastric cancer, Early relapse, Docetaxel, S-1, Irinotecan, Ramucirumab

## Abstract

**Background:**

Although docetaxel plus S-1 adjuvant chemotherapy after gastrectomy with D2 lymphadenectomy has been a standard of treatment for stage III gastric cancer, there is no established chemotherapy for patients with recurrence during or within six months after the completion of adjuvant docetaxel plus S-1 therapy.

**Methods:**

The OGSG 1901 trial is a prospective, open-label, multicenter, phase II trial evaluating ramucirumab plus irinotecan for gastric cancer patients with early relapse after adjuvant docetaxel plus S-1 therapy. The key eligibility criteria were: 1) histologically confirmed gastric adenocarcinoma 2) patients who were on docetaxel plus S-1 adjuvant chemotherapy after the confirmation of pathological stage III, 3) patients with early relapse, i.e., recurrence during or within 6 months after the completion of docetaxel plus S-1 therapy, and 4) patient with Eastern Cooperative Oncology Group performance status of 0–1. Irinotecan (150 mg/m^2^, day 1) and ramucirumab (8 mg/kg, day 1) will be administered every 2 weeks. The primary endpoint is overall survival, and the secondary endpoints are overall response rate, progression-free survival, and safety. The number of patients has been set at 40 based on the threshold and expected median survival times of 7 and 11 months, respectively, with a one-sided alpha error of 0.05 and power of 0.80. The enrollment and follow-up periods are 2 and 1.5 years, respectively.

**Discussion:**

The results of this trial will indicate whether the ramucirumab with irinotecan regimen has the potential to be a recommended treatment regimen for patients with recurrence gastric cancer during or within 6 months after the completion of adjuvant docetaxel plus S-1 therapy.

**Trial registration:**

This study was registered in the Japan Registry of Clinical Trials (jRCTs05119071, October 6, 2019).

## Background

Gastric cancer (GC) is the fifth most common cancer site and the third leading cause of cancer-related deaths worldwide [1]. Although the curative treatment of locally advanced GC is surgery, some cases experience relapse even after R0 resection [2]. Postoperative adjuvant chemotherapy was confirmed to improve relapse-free survival in patients with stage II or III GC who underwent curative gastrectomy. S-1 monotherapy and capecitabine in combination with oxaliplatin have been considered the standard postoperative adjuvant chemotherapy after curative resection in patients with stage II or III GC in Japan [3, 4].

Recently, the JACCRO GC-07 trial showed the superiority of adjuvant docetaxel plus S-1 (DS) over S-1 monotherapy in patients with pathological stage III GC after gastrectomy with D2 lymphadenectomy in terms of 3-year relapse-free survival (RFS) [5], resulting in the establishment of the DS regimen as a standard treatment option for adjuvant therapy of stage III GC in Japan. However, approximately 20% of patients in the DS group experienced early relapse after the adjuvant DS therapy in the JACCRO GC-07 trial. Although previous studies indicated that early relapse during postoperative adjuvant chemotherapy or within six months after adjuvant treatment is associated with a poor prognosis, no prospective clinical trial has evaluated chemotherapy regimen specifically for patients in whom adjuvant DS therapy failed and the optimal treatment of early relapse.

For GC patients with early relapse after postoperative adjuvant therapy, second-line treatment regimens have been adopted [6, 7], given that most of the drugs used in postoperative adjuvant chemotherapy overlap with those used in first-line therapy. Currently, ramucirumab (RAM), a fully human monoclonal antibody that selectively targets vascular endothelial growth factor receptor-2, plus paclitaxel, is the standard second-line treatment [8, 9]. It is thus preferably used for GC patients who experience relapse during or within six months afte adjuvant chemotherapy such as S-1 or capecitabine with oxaliplatin (CapeOX). However, in patients with early relapse after postoperative adjuvant DS therapy, the efficacy of RAM plus paclitaxel is likely limited, given that such tumors are considered taxane-resistant.

Irinotecan (IRI) is another treatment choice for GC. Although IRI is once considered a candidate for second-line chemotherapy, the utility of IRI monotherapy has been shown to be limited compared to that of paclitaxel [10–12]. Thus, attempts are being made to enhance the efficacy of IRI by combining it with RAM for GC treatment, given that RAM plus leucovorin calcium, 5-fluorouracil, and irinotecan (FOLFIRI) has shown efficacy superior to that of FOLFIRI alone [13]. Thus, it has also been established as a standard second-line therapy for colorectal cancer. A Japanese phase Ib trial of RAM plus IRI regimen in more heavily pretreated patients with GC showed that the regimen was safe with sufficient antitumor activity [14]. Recently, the phase II HGCSG 1603 trial demonstrated the efficacy of RAM plus IRI as a second-line therapy for advanced GC patients [15]. In addition, a phase III study evaluating the efficacy of RAM plus IRI as a third-line treatment in patients showing progression after RAM treatment for GC (RINDBeRG, NCT03141034) is ongoing [16]. Considering these facts, we planned a phase II trial to evaluate the efficacy and safety of RAM plus IRI in terms of overall survival among pathological stage III GC patients with recurrence during or within six months after adjuvant DS therapy.

## Methods/design

### Objective

This trial was aimed at exploring and evaluating the safety and efficacy of RAM plus IRI as a second-line therapy for GC patients with early relapse during or after adjuvant DS therapy. The primary endpoint is overall survival (OS). The secondary endpoints are progression-free survival (PFS), overall response rate (ORR), disease control rate (DCR), time-to-treatment failure (TTF), relative dose intensity, and safety. Adverse events will be graded according to the Common Terminology Criteria for Adverse Events (Japanese edition, JCOG version v5.0).

### Study design

**Fig. 1:**
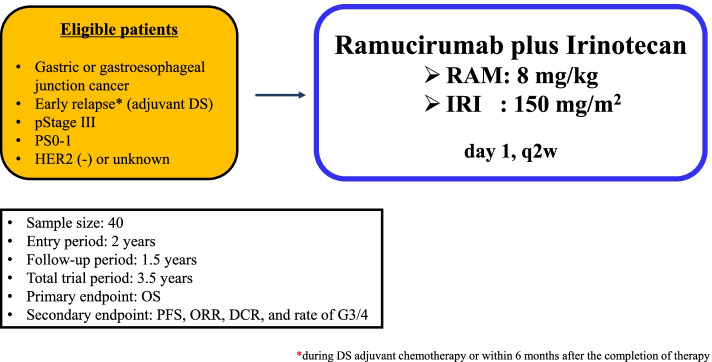
Study schema of the OGSG1901 trial

The OGSG1901 trial is an open-label, prospective, multicenter phase II trial that will be conducted at 25 centers of the Osaka Gastrointestinal Cancer Chemotherapy Study Group (OGSG) in Japan (Fig. 1). The definition of early relapse after surgery is as follows: i) recurrence during DS adjuvant chemotherapy (after at least one docetaxel dose has been administered) and ii) recurrence within 6 months after the completion of DS adjuvant therapy. The study was registered in the Japan Registry of Clinical Trials (jRCTs05119071, October 25, 2019).

### Eligibility criteria

To be eligible for the trial, patients must meet the following criteria:

1. Patients with histopathologically confirmed adenocarcinoma of the gastric or gastroesophageal junction.
2. Patients who are on DS postoperative adjuvant therapy because of confirmed pathological stage III after gastrectomy with D2 lymphadenectomy.
3. Patients with early relapse (during DS postoperative adjuvant therapy or within six months after the completion of DS adjuvant therapy).
4. Patients aged ≥20 years.
5. Patients with Eastern Cooperative Oncology Group (ECOG) performance status (PS) of 0 or 1 at study entry.
6. Patients with lesions that can evaluated on computed tomography (CT) (measurable or non-measurable lesions according to Response Evaluation Criteria in Solid Tumours [RECIST] ver. 1.1).
7. Patients with adequate organ function, defined as no severe disfunction of the lungs, or kidneys, heart, bone marrow, and clinical laboratory test value at the start of protocol therapy that meet the eligibility criteria.
  1. Absolute neutrophil count: ≥1500/mm^3^
  2. Hemoglobin: ≥9.0 g/dL
  3. Platelet count: ≥10.0 × 10^4^/mm^3^
  4. Alanine aminotransferase and aspartate aminotransferase level: ≤100 IU/L (≤200 IU/L in patients with liver metastasis)
  5. Total bilirubin level: ≤1.5 mg/dL (patients with elevated bilirubin associated with grade 1 Gilbert’s syndrome will be exempted)
  6. Serum creatinine of ≤1.5 mg/dL or creatinine clearance of ≥40 mL/min (creatinine clearance rate estimated using the Cockcroft-Gault formula or creatinine concentration measured after 24-h urine collection)
  7. Urinary protein: ≤ (1+) on dipstick or ≥ 2 g/24 h after 24-h urine collection, or urinary protein/creatinine ratio of < 2
  8. Prothrombin time-international normalized ratio (PT/INR): ≤1.5
8. Patients who are fully informed about the trial prior to registration.
9. Patients with an unknown or negative HER2 status.
10. Patients without a history of treatment with IRI or RAM
11. Patients with a history of chemotherapy or chemoradiotherapy prior to surgery

### Exclusion criteria

Patients who meet any of the following criteria are not eligible for the study.

1. Patients with active double primary malignancies.
2. Patients with symptomatic interstitial pulmonary fibrosis or pneumonia.
3. Patients with an active infection.
4. Patients with a serious illness or medical condition.
5. Patients who are possibly pregnant, pregnant, unwilling to practice contraception during the study or breastfeeding.
6. Patients diagnosed with mental illness or with manifestations of mental illness indicating that they could have difficulty in participating in the trial.
7. Patients on systemic steroids.
8. Patients with a serious complication.
  1. A history of pulmonary embolism, active vein thrombosis, and other serious thromboembolism.
  2. A history of unstable angina pectoris, myocardial infarction, transit ischemic attack, or cerebral apoplexy within 6 months prior to registration.
  3. A history of gastrointestinal fistula or perforation prior to registration.
  4. A history of impaired wound healing, bone fructure or ulcer within 4 weeks prior to registration.
  5. A major surgery, such as laparotomy, scheduled within 28 days of the first drug administration or percutanous surgery as well as other minor surgery scheduled within 7 days of the first drug administration.
  6. Child–Pugh score of B or above for severity of cirrhosis, hepatic encephalopathy, or ascites fluid accumulation requiring treatment.
9. Patients receiving anticoagulants for thromboembolism treatment.
10. Patients with uncontrolled hypertension.
11. Patients with uncontrolled diarrhea.
12. Patients showing symptomatic evidence of known central nervous system metastases.
13. Patients who receive blood transfusion treatment within 2 weeks of registration.
14. Patients with moderate pleural effusion and ascites.
15. Patients on ataznavir sulfate.
16. Other situations in which the trial investigator determines that the subject is an unsuitable candidate.

### Treatment

Patients will receive RAM (8 mg/kg) and IRI (150 mg/m^2^) intravenously on day 1 every 2 weeks. Although the *UGT1A1* test is not mandatory, in patients who are known to have homozygous or doubly heterozygous polymorphisms in the *UGT1A1* gene, irinotecan administration will be initiated with a reduction in dose level 1 (120 mg/m^2^). If grade 3 or higher hematological toxicity, or grade 3 non-hematological toxicity with a causal relationship with IRI is recorded, the IRI dose must be reduced to the next level. If fatal RAM-related adverse events or grade 3 infusion reactions are observed, RAM must be discontinued. If subjects show ≥2+ on a dipstick proteinuria or if grade 3 adverse events are recorded, the RAM dose must be reduced to the next levwl. If subjects show a grade 4 hypertension, grade 3 or higher infusion reaction or ≥ 3 g/24 hour proteinuria, RAM must be discontinued (Table 1). The protocol regimen repeat every 14 days until unacceptable toxicity, disease progression, or consent withdrawal. The palliative radiotherapy and treatment with other anticancer drugs will be not allowed.

**Table 1:** Decreased dosage level

Decreased dosage level	Irinotecan	Ramucirumab
-1	120 mg/ m^2^	6 mg/kg
-2	100 mg/m^2^	5 mg/kg
-3	80 mg/m^2^	0 mg/m^2^

### Outcomes

Disease assessment is performed every 8 weeks using computed tomography (CT) and/or magnetic resonance imaging (MRI). The ORR is defined as the percentage of patients relative to the total number of enrolled subjects who achieved a complete response (CR) or partial response (PR) based on imaging. The ORR assess according to Response Evaluation Criteria in Solid Tumors (RECIST) version 1.1. PFS is defined as the time from enrollment to disease progression or death. OS is defined as the time from enrollment to death. The assess according to the Common Terminology Criteria for Adverse Events version 5.0.

### Study design and statistical considerations

An independent review member will assess treatment efficacy and safety. Based on the WJOG4007 study and multicenter retrospective study, the null hypothesis is “OS is 7.0 months,” and the alternative hypothesis is “OS is >11 months”. The following hypothesis will be assessed using the exact binomial test of one-sided alpha level of 0.05 and a power of 0.90 were calculated using the Clopper-Pearson exact method [17]. The sample size calculated to 39. Considering dropouts, a total of 40 patients were enrolled. The enrollment period is 2 years. The follow-up period is 1.5 years from the last enrollment.

### Monitoring

The monitoring of study group (OGSG) will independently review data of safety and efficacy obtained in this study. Trial monitoring will be performed annually. Accordingly, the monitoring committee will determine the modification of the study protocol and early termination of a treatment regimen. Moreover, safety, protocol compliance, and trial progress will be monitored by the monitoring committee.

## Discussion

The 2018 edition of the GC Guideline in Japan does not recommend the use of chemotherapy agents that have already been administered in the postoperative adjuvant chemotherapy to treat malignancy that has early relapsed [6]. This recommendation is, in part, based on the retrospective study by Shitara et al. They found that S-1 plus cisplatin was ineffective in patients with recurrent GC, especially with a relapse-free interval (RFI) of less than six months after postoperative adjuvant S-1 chemotherapy [7]. Furthermore, the survival benefit of cisplatin with S-1 was less in patients with RFI of < 6 months compared to that in those with RFI of ≥6 months, with a median PFS of 6.2 vs. 2.3 months and a median OS of 16.6 vs. 7.3 months, respectively.

Considering the increased use of DS adjuvant postoperative chemotherapy for pathological stage III GC, the clinical issue of recurrence after DS is becoming increasingly important. Although the RAM plus paclitaxel regimen is the standard second-line treatment and thus preferably used in cases of early relapse, continuous treatment with a taxane-containing regimen after DS therapy may not be the preferred treatment option, raising the need for developing non-taxane regimens. In this regard, IRI can be an important therapeutic choice as a non-taxane-containing regimen for patients who have failed adjuvant DS therapy. It was assumed that the patients eligible for this study would most likely not have measurable lesions. In that case, PFS is not the appropriate primary endpoint, given the difficulty to determine disease progression based on imaging, which affects the reliability of this endpoint. We thus adopted OS as the primary endpoint although a single-arm phase II study.

Neoadjuvant strategies adopt in Western countries, especially in Europe, where the 5-fluorouracil plus leucovorin plus oxaliplatin docetaxel (FLOT) regimen showed significant survival benefits compared with the cisplatin plus epirubicin plus continuous infusion 5-fluorouracil regimen for patients with locally advanced GC [18]. The FLOT regimen has been adopted as a first-line chemotherapy for patients with advanced GC. However, detailed information about disease progression or early disease recurrence after FLOT regimen in the first-line or perioperative setting has not been reported, raising need for an another chemotherapy regimen in 2nd-linet chemotherapy regimen other than paclitaxel [19], given the possibility of cross-resistance between paclitaxel and docetaxel. Against this background, the phase II RAMIRIS trial (NCT03081143) was conducted to compare RAM plus FOLFIRI with paclitaxel plus RAM as a second-line treatment for patients with advanced gastroesophageal cancer, including those showing progression after FLOT treatment [20]. The trial demonstrated promising efficacy of FOLFIRI plus RAM vs. paclitaxel plus RAM in the FLOT-pretreated subgroup with an ORR of 25% vs. 8%, and median PFS of 4.6 and 2.1 months, respectively. Accordingly, the FOLFIRI plus RAM regimen is regarded as one of the optimal “second-line” taxane-free regimens for docetaxel-pretreated GC patients.

However, the effectiveness of fluoropyrimidine re-administration beyond the first disease progression after fluoropyrimidine-based regimens is unclear. As mentioned above, the cisplatin with S-1 regimen has limited efficacy in patients with early relapse after adjuvant S-1 therapy [7]. In a phase II study of cisplatin with capecitabine for patients with GC who showed recurrence within or more than six months after postoperative chemotherapy with fluoropyrimidine-based regimens [21], the ORR (39 vs. 21%, *P* = 0.427), and OS (14.1 vs. 9.3 months, *P* = 0.075) tended to be worse in patients with an RFI of ≤6 months than in patients with an RFI of > 6 months. These findings support the use of IRI + RAM, not FOLFIRI+RAM, in this setting. Furthermore, in Japan, most chemotherapeutic regimens for advanced GC do not involve the use of peripheral venous access ports. The RAM plus IRI regimen, the port-free regimen, is thus a more reasonable option than FOLFIRI in Japan.

There, the RAM plus IRI regimen is expected to prolong the OS of GC patients with early relapse during or after DS adjuvant chemotherapy. We conducted a phase II trial to investigate the efficacy of RAM plus IRI in patients with early recurrence who were refractory to fluoropyrimidine and docetaxel. The results of this study will help establish treatment options for GC patients with early relapse after DS therapy.

## Data Availability

Not applicable.

## Abbreviations

CapeOX: Capecitabine Plus Oxaliplatin
CI: Confidence Interval
CT: Computed Tomography
DCR: Disease Control Rate
DS: Docetaxel plus S-1
DSMC: Data and Safety Monitoring Committee
FOLFIRI: Leucovorin calcium, 5-fluorouracil, and irinotecan
GC: Gastric Cancer
HR: Hazard Ratio
IRI: Irinotecan
OGSG: Osaka Gastrointestinal Cancer Chemotherapy Study Group
ORR: Overall Response Rate
OS: Overall Survival
PFS: Progression-Free Survival
PS: Performance Status
RAM: Ramucirumab
RECIST: Response Evaluation Criteria in Solid Tumours
RFI: Relapse-Free Interval
RFS: Relapse-Free Survival
TTF: Time-to-Treatment Failure
